# Characterization of age-related immune features after autologous NK cell infusion: Protocol for an open-label and randomized controlled trial

**DOI:** 10.3389/fimmu.2022.940577

**Published:** 2022-09-29

**Authors:** Xiaofeng Tang, Biaolong Deng, Aiping Zang, Xiaowen He, Ye Zhou, Daimeng Wang, Dan Li, Xueyu Dai, Jieqiong Chen, Xuhua Zhang, Ye Liu, Yonghua Xu, Jingjing Chen, Weijie Zheng, Luding Zhang, Constance Gao, Huanfeng Yang, Bin Li, Xueqi Wang

**Affiliations:** ^1^ Department of Blood Transfusion, Changzheng Hospital, Shanghai, China; ^2^ Department of Immunology and Microbiology, Shanghai Institute of Immunology, Shanghai Jiao Tong University School of Medicine, Shanghai, China; ^3^ Department of Research and Development, Shanghai Origincell Medical Technology Co., Ltd., Origincell Technology Group Co., Ltd., Shanghai, China; ^4^ Department of Research and Development, Shanghai Affinity Biopharmaceutical Co., Ltd., Shanghai, China; ^5^ Department of Biology, College of Science, Northeastern University, Boston, MA, United States

**Keywords:** aging, T-cell senescence, T-cell exhaustion, natural killer cells, SASP

## Abstract

**Background:**

Aging is usually accompanied by functional declines of the immune system, especially in T-cell responses. However, little is known about ways to alleviate this.

**Methods:**

Here, 37 middle-aged healthy participants were recruited, among which 32 were intravenously administrated with expanded NK cells and 5 with normal saline. Then, we monitored changes of peripheral senescent and exhausted T cells within 4 weeks after infusion by flow cytometry, as well as serum levels of senescence-associated secretory phenotype (SASP)-related factors. *In vitro* co-culture assays were performed to study NK-mediated cytotoxic activity against senescent or exhausted T cells. Functional and phenotypic alteration of NK cells before and after expansion was finally characterized.

**Results:**

After NK cell infusion, **s**enescent CD28^-^, CD57^+^, CD28^-^CD57^+^, and CD28^-^KLRG1^+^ CD4^+^ and CD8^+^ T-cell populations decreased significantly, so did PD-1^+^ and TIM-3^+^ T cells. These changes were continuously observed for 4 weeks. Nevertheless, no significant changes were observed in the normal saline group. Moreover, SASP-related factors including IL-6, IL-8, IL-1α, IL-17, MIP-1α, MIP-1β, and MMP1 were significantly decreased after NK cell infusion. Further co-culture assays showed that expanded NK cells specifically and dramatically eliminated senescent CD4^+^ T cells other than CD28^+^CD4^+^ T cells. They also showed improved cytotoxic activity, with different expression patterns of activating and inhibitory receptors including NKG2C, NKG2A, KLRG1, LAG3, CD57, and TIM3.

**Conclusion:**

Our findings imply that T-cell senescence and exhaustion is a reversible process in healthy individuals, and autologous NK cell administration can be introduced to alleviate the aging.

**Clinical Trial Registration:**

ClinicalTrials.gov, ChiCTR-OOh-17011878.

## Introduction

Aging is characterized by a progressive loss of physiological function, and is a risk factor for several of the world’s most prevalent diseases (1–3). According to the World Health Organization, there will be more than 30% of the population aged 60 years or older in China, America, and several European countries by 2050 (https://www.who.int/ageing/en/), and the incidence of related diseases, including cancer, T2DM (type 2 diabetes), neurodegenerative disorders, and cardiovascular disease, will increase with aging.

Normally, aging is associated with a progressive decline in the function of immune system, among which natural killer (NK) cells and T cells are key components in innate and adaptive immunity, respectively (4, 5). NK cells, characterized by expressing CD16 and CD56, play critical roles as the first line of defense against virus infection and cancer cells (6, 7). Young individuals have high levels of functional NK cells. However, the NK-related activities decline with aging, leading to an increased incidence and severity of viral infections (8, 9). In addition, Liu et al. have found that primary NK and CAR-NK cells have superior expansion capability and *in vivo* cytotoxicity after optimizing the cultural condition, which broaden cell therapy application (10). Moreover, due to repeated antigenic stimulation throughout life, aging is always accompanied by increased accumulation of senescent and exhausted T cells, which, in turn, leads to impaired T cell-mediated responses (11). This decline is largely responsible for the increased susceptibility to infection, reduced effectiveness of vaccination, and higher incidences of diseases including cancer in the elderly (12).

Recent findings from several clinical studies have shown that markers of T-cell senescence (i.e. the loss of CD28 and/or gain of CD57 among CD4^+^/CD8^+^ T-cell) (13, 14) and T-cell exhaustion (i.e. high expression of PD-1 among CD4^+^/CD8^+^ T-cell) (15) are usually higher in patients with HIV infection, breast cancer or myeloid leukemia (AML) than in healthy controls (16). Of note, the population and/or numbers of senescent and exhausted T cells are reversed following anti-viral treatment and chemotherapy (17–19). Importantly, these declining changes are mostly restricted to complete remission patients other than non-responders, implying that they are highly predictable and positively related to clinical outcomes (20). Natural killer cells play critical roles in immune clearance of aging-related senescent cells, which may modulate T-cell dysfunction (21, 22). However, whether NK cells could boost immune system in sub-health population is still unknown.

Accumulation of senescent cells in aging may promote immune senescence by developing a senescence-associated secretory phenotype (SASP) and generating damage signals (23, 24). Given the critical roles of NK cells in immune clearance of senescent cells and their declining activities with aging (25), here we firstly explored whether the administration of autologous NK cells would affect the peripheral population of senescent and exhausted T cells in middle-aged healthy individuals. Accordingly, the CD28^-^, CD57^+^, CD28^-^CD57^+^, and CD28^-^KLRG1^+^ expressing CD4^+^ and CD8^+^ T cells were assessed as senescent T cells, while the PD1^+^ and TIM3^+^ expressing CD4^+^ and CD8^+^ T cells were assessed as exhausted T cells here. Results showed that senescent T cells, including CD28^-^, CD57^+^, CD28^-^CD57^+^, and CD28^-^KLRG1^+^ subsets, decreased significantly in both CD4^+^ and CD8^+^ T cells following a single infusion of autologous NK cells. In addition, the PD1^+^ and TIM3^+^ population within CD4^+^ and CD8^+^ T cells also dramatically declined after the infusion. Declines were continuously observed in senescent and exhausted T cells for 4 weeks after the intervention. Meanwhile, the concentrations of chemokines, inflammatory cytokines, tumor necrosis factors, and growth factors in the serums were assayed. We found that SASP-related factors including IL-6, IL-8, IL- 1α, IL-17, MIP-1α, MIP-1β, and MMP1 were significantly decreased after NK cell infusion. Of note, expanded NK cells dose-dependently reduced the population of CD28^-^ and CD28^-^CD57^+^ CD4^+^ T cells *in vitro* during co-culture assay, strongly suggesting NK cells’ ability to recognize and remove senescent cells. They also showed improved cytotoxic activity against K562. Furthermore, different expression patterns of activating and inhibitory receptors including NKG2C, NKG2A, KLRG1, LAG3, CD57, and TIM3 were observed after *in vitro* expansion procedure, probably contributing to their functional alteration.

## Methods

### Subjects

This study (ClinicalTrials.gov identifier: ChiCTR-OOh-17011878) was approved by the Ethical Committee of Changzheng Hospital. Subjects were eligible for this study if they were 45–55 years old and disease-free. Subjects with a positive serology for human immunodeficiency virus (HIV), hepatitis B virus (HBV), hepatitis C virus (HCV), Epstein–Barr virus (EBV), cytomegalovirus (CMV), and syphilis were excluded, as were those with two or more abnormal testing results in the liver function tests including alanine aminotransferase (ALT), aspartate aminotransferase (AST), total bilirubin (TBil), indirect bilirubin (I-TBil), direct bilirubin (DBIL), and γ-GT. Also, subjects with tumor marker alpha fetoprotein (AFP) and carcinoembryonic antigen (CEA) were excluded.

### Study description

All subjects had physical examinations and medical questionnaires to assess the health status. Then, eligible subjects signed the informed consent form before entering the group where they received a dose of autologous NK cells in two infusions in 2 days. Peripheral blood samples were collected before cell infusion as baseline and at the first and fourth weeks after cell infusion to evaluate the effects of NK cell administration on T-cell senescence and exhaustion, as well as SASP.

### *In vitro* natural killer cell amplification and cell infusion

Leukapheresis was carried out to collect peripheral blood mononuclear cells (PBMCs) from subjects by Spectra Optia (TERUMO, USA). NK cells from fresh or cryopreserved PBMCs were amplified *in vitro* using a feeder cell free culture system with the Natural killer cells culture kit (DAKEWE, China). In brief, cells were seeded into activator-coated flasks at 1–2 × 10 (6) cells/ml and incubated in a 37°C–5% CO_2_ incubator (Thermo fisher, USA). Fresh NK medium was changed every 2–3 days until sufficient amounts of cells were obtained about 14 days later. Quality control was conducted by assessing samples taken during the entire culture period and the final cell product. The BacT/ALERT (bioMerieux, Durham, NC, USA) microbiological detection system was used for sterility, and the gel-clot technique using amoebocyte lysate from the horseshoe crab was used for endotoxin test. Mycoplasma contamination tests were performed by PCR method using specific primers of mycoplasma (Yise Medical, China). Trypan staining was used to calculate the number and viability of NK cells. NK cells, determined by the expression of CD56 or CD16 and the absence of CD3 (**Supplementary Figure 1A**), were quantified using flow cytometry with antibodies purchased from BD Biosciences including anti-CD3 antibody (HIT3, FITC), anti-CD56 antibody (B159, APC), and anti-CD16 antibody (B73.1, APC) (26). We also determined the population of C56^-^CD16^+^CD3^-^ in expanded NK cells, and it turned out that less than 2% of this subset was contained, as indicated in one representative result out of three individual expanded NK cells in **Supplementary Figure 1B**. NK cells were resuspended in saline solution containing human serum albumin and were intravenously injected into subjects at the first drip rate of 1 ml/min following 2–3 ml/min in two equal lots in 2 days. At the end of every infusion, another 60–70 ml of saline solution was used to flush the pipeline of the disposal transfusion set.

### Immuno-phenotypic analysis of peripheral T cells

PBMCs were isolated using Ficoll-Paque (GE Healthcare, USA), and the cellular phenotypic of senescent and exhausted T cells was analyzed by flow cytometry. For surface markers analysis, cells were stained in PBS containing 2% fetal bovine serum (FBS, Thermo fisher, USA) with antibodies as indicated. Then, flow cytometric analysis was carried out in BD LSRFortessa X20. The gating strategy to identify T cell subsets was applied as described previously (27). Antibodies used in this study were purchased from BD Biosciences and eBioscience, including anti-CD4 (GK1.5, 1:100), anti-CD8 (53-6.7, 1:100), anti-CD25 (PC61, 1:100), anti-CD45RA (HI100, 1:100), anti-CXCR3(G025H7, 1:100), anti-CCR4 (L291H4, 1:100), anti-CCR6 (G034E3, 1:100), anti-CCR7 (G043H7, 1:100), anti-CD127 (A019D5, 1:100), anti-CXCR5 (RF8B2, 1:100), anti-CD28 (CD28.2, 1:100), anti-CD57 (NK-1, 1:100), anti-KLRG1 (2F1, 1:100), anti-PD-1 (EH12.2H7, 1:100), anti-TIM3 (F38-2E2, 1:100), and fixable viability dye eFluor 780 (eBioscience, Cat#65-0865-14, 1:1,000).

### Cytokine determination

Cytokines, MMP-1, MIP-1β, MIP-1α, IL-8, IL-1α, IL-6, IL-17A, and IFN-γ, in blood plasma were detected by Luminex xMAP technology with the multiplex assay kit (ProcartaPlex 8 Plex, Thermo Fisher, PPX-08).

### *In vitro* co-culture assay of NK cells with PBMCs or senescent and exhausted T cells

To determine the effect of expanded NK cells on senescent T cells, PBMCs were harvested, washed, counted, and diluted to 5 × 10 (4) cells/ml, and 50 μl/well were plated in a 96-well plate. NK cells were washed, counted, diluted, and added at an NK:PBMC cell ratio of 10:1, 1:1, and 0:1. All of the conditions were assayed in quadruplicate. After 24 h at 37°C, cells were washed and stained for flow cytometry analysis of variation of dead CD4^+^CD28^+^ and CD4^+^CD28^-^ cells with anti-CD4 (GK1.5, 1:100), anti-CD28 (CD28.2, 1:100), and fixable viability dye eFluor 780 (eBioscience, Cat#65-0865-14, 1:1,000).

To determine specific cytotoxicity, CD28^-^CD57^+^CD4^+^ or CD28^+^CD4^+^ T cells were sorted from PBMCs. Antibodies used in this study were from BD Biosciences and eBioscience, including anti-CD4 (GK1.5, 1:100), anti-CD28 (CD28.2, 1:100), anti-CD57 (NK-1, 1:100), and fixable viability dye eFluor 780 (eBioscience, Cat#65-0865-14, 1:1,000). Then, we used the CytoTox 96 Nonradioactive Cytotoxicity assay (Pro-mega) based on the calorimetric detection of the released enzyme LDH (28). Target cells were harvested, washed, counted, and diluted to 5 × 10 (4) cells/ml, and seeded at 50 μl/well in a 96-well plate. Lymphocytes were washed, counted, diluted, and added at an effector:target cell ratio of 10:1 and 1:1. All of the conditions were assayed in quadruplicates. After 24 h co-culture at 37°C, 50 μl of supernatants was assayed for LDH activity following the manufacturer’s protocol. Controls for spontaneous LDH release in effector and target cells, as well as target maximum release, were prepared. The calculation of cytotoxicity percentage was performed as follows:


% cytotoxicity=[Experimental−effector spontaneous−Target spontaneous]/[Target maximum−target spontaneous] × 100

Only targets with spontaneous release of LDH ≤10% of the maximum release were considered.

Additionally, CD3^+^PD-1^+^ T cells were sorted from PBMCs with antibodies of anti-CD3 (HIT3, 1:100) and anti-PD-1 (NAT105, 1:100), and used as target cells in co-culture with NK cells sorted from PBMC (indicated as preNK cells) and expanded NK cells (indicated as postNK cells) at an effector-to-target (E:T) cell ratio of 10:1. After 20 h of incubation at 37°C, both LDH assay described as before and flow cytometry analysis were carried out to determine NK-mediated cytotoxicity against exhausted T cells.

### Cytotoxicity of preNK and postNK

K562 cells with stable expression of luciferase were used as target cells and co-cultured with preNK or postNK at an E:T ratio of 10:1. After 18 h of incubation, luciferase activity was determined according to Luciferase assay system TB281 (Promega). Target maximum luciferase activity was prepared. The calculation of cytotoxicity percentage was performed as follows:

% cytotoxicity = [Target maximum − Experimental]/Target maximum] × 100%. Meanwhile, supernatants were collected to determine NK-released perforin and IFNγ with the human perforin ELISA kit (SEKH-0295, Solarbio) and the human IFNγ ELISA kit (DY285, R&D).

### Phenotype of preNK and postNK

Flow cytometry analysis was carried out to study phenotype variation of NK cells before and after *in vitro* expansion. Antibodies used here were from BD Biosciences and eBioscience, including anti-NKG2C (134591, 1:100), anti-NKG2A (131411, 1:100), anti-LAG3 (11C3C65, 1:100), anti-KLRG1 (14C2A07, 1:100), anti-CD57 (HNK-1, 1:100), and anti-TIM3 (7D3, 1:100).

### Statistical analysis

Data in this study were analyzed in GraphPad Prism 7.0, and represented as means ± SEM (the standard error of the mean) or means ± SD. The statistical significance was determined by one-way ANOVA for multiple comparisons. *p*-values were denoted in figures in the following way: ns: not significant; *p < 0.05; **p < 0.01; ***p < 0.001; ****p < 0.0001. Power calculation was carried out in analyzing saline-related effects on senescent and exhausted T cells, with alpha set at 0.05.

## Results

### Baseline characteristics

**Figure 1:**
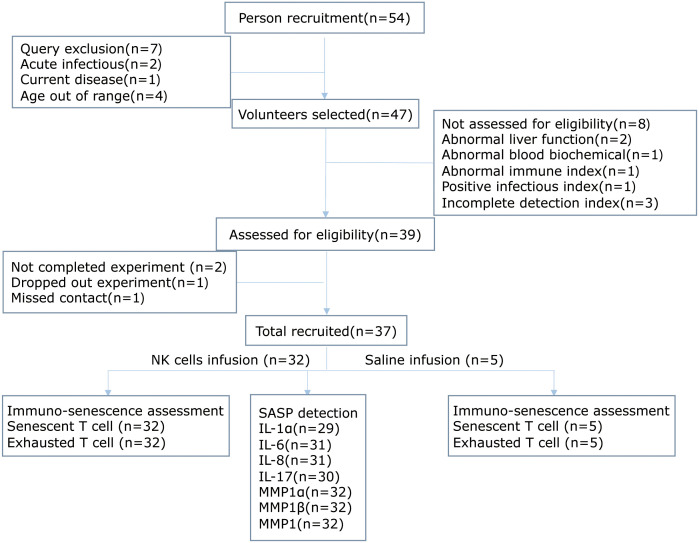
The study flowchart in line with the STROBE.

From July 2017 to September 2018, 47 out of a total of 54 subjects aged from 45 to 55 years old were recruited into the study, because these middle-aged populations were reported to have impaired biology of NK cells (29, 30). During screening, eight volunteers were excluded, among which three had incomplete detection index, two had two or more abnormal results in liver function tests, one had abnormal immune index, one had positive infectious index, and one had abnormal blood biochemical index. Therefore, 39 volunteers were enrolled in this study. They received leukapheresis and subsequent NK cell administration. However, two subjects missed their sample collection after NK cell administration and had to exit from the study. Finally, 37 subjects successfully completed the study, including 18 male and 19 female subjects (**Figure 1**). Importantly, all the volunteers consulted and signed the informed consent form before participation. Meanwhile, 32 volunteers were re-injected with autologous NK cells, while 5 were administrated with normal saline. The baseline characteristics of the subjects and NK cell information are listed in **Supplementary Table 1**.

### Safety of autologous NK cell infusion

After NK cell administration, all subjects had normal body temperature and blood pressure. No one developed skin rashes, local infection and bleeding, fever, chills, difficult breathing, nausea, and vomiting. However, one subject developed agrypnia within 1 week after cell infusion and recovered thereafter. One developed dizziness within 1 week after cell infusion, and this phenomenon lasted for 2 weeks before recovering. Two subjects developed fatigue, among which one developed mild fatigue and the other developed media fatigue, and both recovered in 2 weeks (**Supplementary Table 2**). Furthermore, we conducted routine blood test, hematological examination, and urinary and virological examination at 1 month after cell infusion. No hepatotoxicity and nephrotoxicity were observed according to normal serum levels of ALT, AST, urea, and creatinine. Additionally, no abnormal C response protein (CRP), anti-thyroglobulin antibody (TGAb), and anti-thyroid peroxidase autoantibody (TPOAb) activities were observed, indicating that no immune response and autoimmune effects occurred. Furthermore, no increased plasma levels of alpha fetoprotein (AFP) and carcinoembryonic antigen (CEA) were observed 1 month later, strongly confirming that autologous NK cell infusion was safe in terms of tumorigenicity.

### Senescent T cells decreased after NK cell infusion

**Figure 2:**
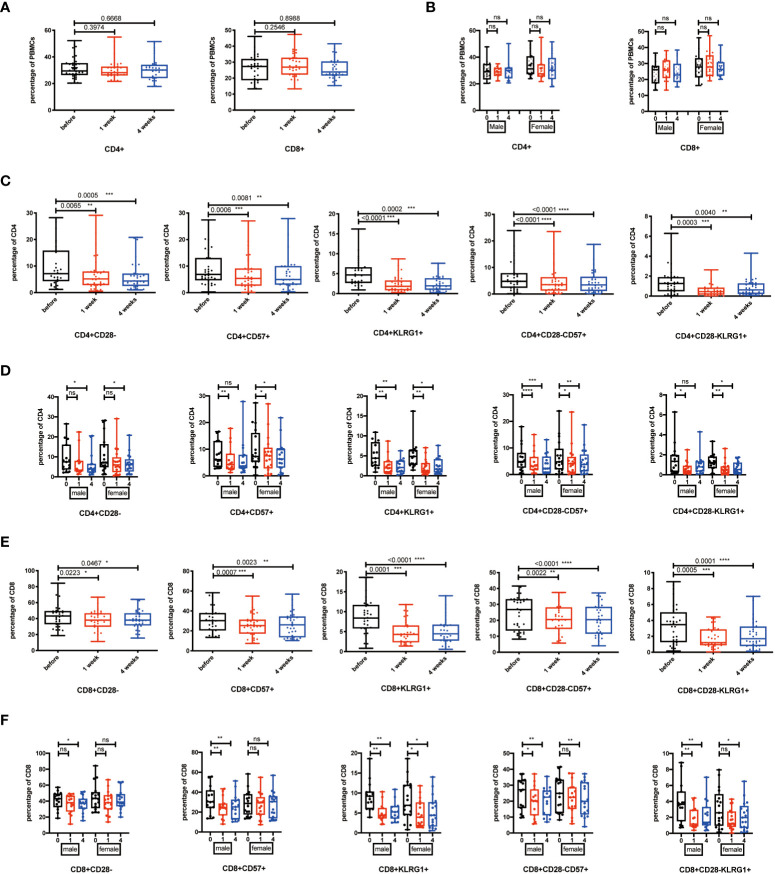
Senescent T cells decreased after NK cell infusion. **(A)** Percentages of CD4^+^ and CD8^+^ T cells were analyzed by flow cytometry on the 1 week and 4 weeks after NK cell infusion**(B)** Percentages of CD4^+^ and CD8^+^ T cells were analyzed in both sex by flow cytometry on the 1 week and 4 weeks after NK cell infusion. **(C)** Percentages of CD28^-^, CD57^+^, KLRG1+, CD28^-^CD57^+^, and CD28^-^KLRG1^+^ CD4^+^ T cells were analyzed by flow cytometry on the 1 week and 4 weeks after NK cell infusion. **(D)** Percentages of CD28^-^, CD57^+^, KLRG1+, CD28^-^CD57^+^, and CD28^-^KLRG1^+^ CD4^+^ T cells were analyzed in both sex by flow cytometry on the 1 week and 4 weeks after NK cell infusion. **(E)** Percentages of CD28^-^, CD57^+^, KLRG1^+^, CD28^-^CD57^+^, and CD28^-^KLRG1^+^ CD8^+^ T cells were analyzed by flow cytometry on the 1 week and 4 weeks after NK cells infusion. **(F)** Percentages of CD28^-^, CD57^+^, KLRG1+, CD28^-^CD57^+^, and CD28^-^KLRG1^+^ CD8^+^ T cells were analyzed in both sex by flow cytometry on the 1 week and 4 weeks after NK cell infusion. Each marker has detected 32 volunteers; 15 volunteers are involved in the male group, and 17 volunteers are involved in the female group. *p < 0.05; **p < 0.01; ***p < 0.001; ****p < 0.0001. ns, no signifcant.

Previous studies have proven that the accumulation of senescent cells accelerates aging-associated disorders, and the clearance of p16-positive cells delays this phenomenon (31). NK cells play important roles in innate immunity for clearing senescent cells and defending against cancer (22). Thus, flow cytometry analysis was carried out to detect populations of CD4^+^CD28^-^, CD4^+^CD57^+^, CD4^+^KLRG1^+^, CD4^+^CD28^-^CD57^+^, and CD4^+^CD28^-^KLRG1^+^ as senescent CD4^+^ T cells at baseline, and 1 and 4 weeks after infusion. Results showed no significant changes in CD4^+^ and CD8^+^ T-cell populations at two time points after cell infusion (**Figures 2A, B**). However, senescent CD4^+^ T cells were significantly decreased 1 and 4 weeks after NK cell infusion (**Figure 2C**). In addition, gender had no impact on the reduction of senescent CD4^+^ T cells caused by NK cell infusion (**Figure 2D**).

As we know, CD8^+^ T cells are the main tumor killing cell group (32). CD8^+^ cytotoxic T cells can attenuate tumor growth by expressing FasL and secreting granzyme B and IFN-γ (33). However, the accumulation of senescent T cells impairs T cell-mediated responses. Thus, we checked the percentage of CD28^-^, CD57^+^, KLRG1^+^, CD28^-^CD57^+^, and CD28^-^KLRG1^+^ senescent CD8^+^ T cells at baseline, and 1 and 4 weeks after infusion. We found out that senescent CD8^+^ T cells significantly decreased, at both 1 week and 4 weeks, after NK cell infusion (**Figure 2E**). The NK cell infusion-induced reduction of senescent CD8^+^ T cells was gender independent (**Figure 2F**).

### Exhausted T cells decreased after NK cell infusion

**Figure 3:**
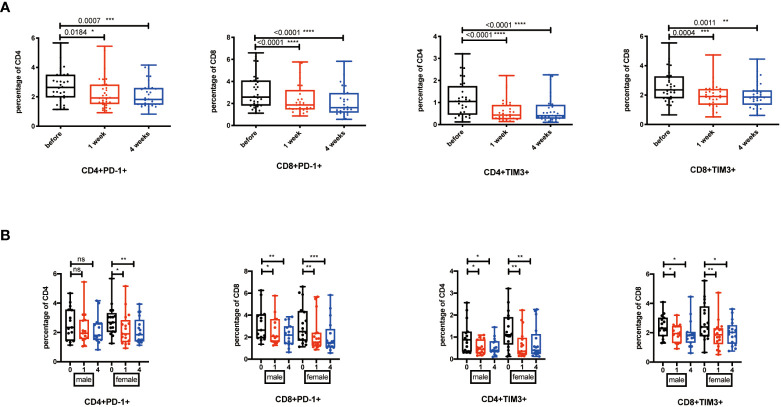
Exhausted T cells decreased after NK cell infusion. **(A)** Percentages of PD-1^+^ and TIM-3^+^ T cells were analyzed by flow cytometry on the 1 week and 4 weeks after NK cell infusion. **(B)** Percentages of PD-1^+^ and TIM-3^+^ T cells were analyzed in both sex by flow cytometry on the 1 week and 4 weeks after NK cell infusion. Each marker has detected 32 volunteers; 15 volunteers are involved in the male group, and 17 volunteers are involved in the female group. *p < 0.05; **p < 0.01; ***p < 0.001; ****p < 0.0001. ns, no signifcant.

During chronic infections and cancer, memory T cells differentiate along with persistent antigen exposure and inflammation (15). It has been reported in human that T-cell exhaustion happens during viral infections, such as HIV and hepatitis C virus (HCV), and cancer development (34, 35). Importantly, exhausted T cells are characterized by elevated expression of PD-1, TIM-3, CTLA-4, and the activation of their related signaling pathways. Recent successful applications of anti-PD-1/PD-L1 antibodies in cancer immunotherapy have proven the significance and efficacy of treatments targeting T-cell exhaustion (36). Then, we detected whether NK cell infusion affected the percentage of exhausted CD4^+^ and CD8^+^ T cells. Results showed that CD4^+^PD-1^+^ T cells, CD8^+^PD-1^+^ T cells, CD4^+^TIM-3^+^ T cells, and CD8^+^TIM-3^+^ T cells were significantly decreased after NK cell infusion at both 1 week and 4 weeks (**Figure 3A**). These results suggested that NK cell infusion might improve the function of T cells by alleviating the exhausted status of T cells. Moreover, the decreases of PD-1^+^ and TIM-3^+^ T cells, at 1 week and 4 weeks after NK cell infusion, were gender independent (**Figure 3B**).

### Cell types influenced the effects induced by NK cell infusion

**Figure 4:**
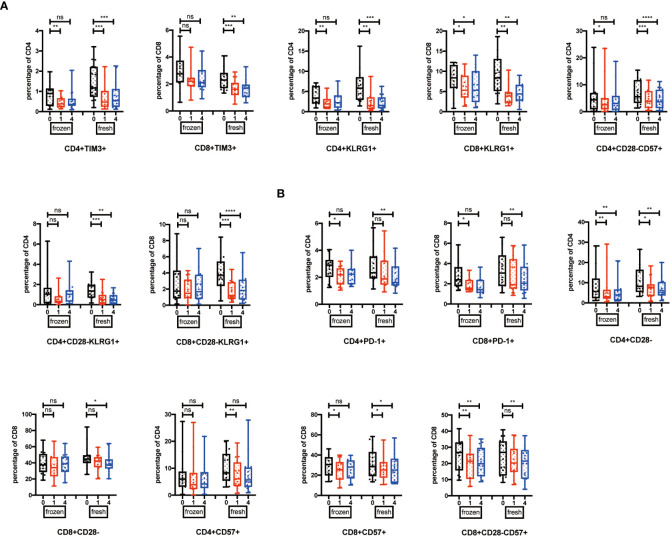
Cell types influence the effects induced by NK cell infusion. **(A)** Percentages of TIM-3^+^, KLRG1+, CD28^-^CD57^+^, and CD28^-^KLRG1^+^ T cells were analyzed in both fresh and frozen groups by flow cytometry on the 1 week and 4 weeks after NK cell infusion. **(B)** Percentages of PD-1^+^, CD28^-^, and CD57^+^ T cells were analyzed in both fresh and frozen groups by flow cytometry on the 1 week and 4 weeks after NK cell infusion. Each marker has detected 32 volunteers; 14 volunteers are involved in the frozen group, and 18 volunteers are involved in the fresh group. *p < 0.05; **p < 0.01; ***p < 0.001; ****p < 0.0001. ns, no signifcant.

Among the 32 participants receiving NK cells, 14 volunteers were re-injected with NK cells amplified from cryopreserved PBMCs, and 18 volunteers were re-injected with NK cells generated from fresh PBMCs. We would like to explore whether the NK cell infusion-induced immune system alterations were dependent on the beginning status of NK cells. We analyzed these two groups by one-way ANOVA for multiple comparisons. The results showed that TIM3^+^, KLRG1^+^, CD28^-^CD57^+^, and CD28^-^KLRG1^+^ T cells were significantly reduced at the 1- or 4-week point in the fresh NK cell infusion group (**Figure 4A**), while CD28^-^, CD57^+^, and PD-1^+^ T cells did not significantly change in the fresh NK cell infusion group compared with frozen NK cell infusion (**Figure 4B**).

### Key SASP-related factors reduced after NK cell infusion

**Figure 5:**
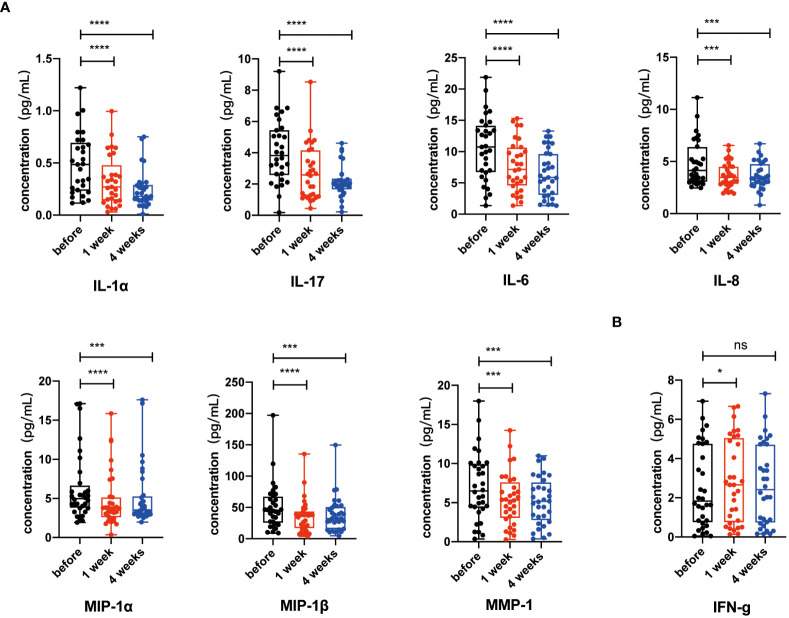
Key senescence-associated secretory phenotype (SASP) components decreased after NK cell infusion. **(A)** IL-6, IL-8, IL-1α, IL-17, MIP-1α, MIP-1β, and MMP1 were detected by Luminex xMAP technology on the 1 week and 4 weeks after NK cell infusion. **(B)** IFN-γ were detected by Luminex xMAP technology on the 1 week and 4 weeks after NK cell infusion. Data [IL-6 (*n* = 31), IL-8 (*n* = 31), IL-1α (*n* = 29), IL-17 (*n* = 30), MIP-1α (*n* = 32), MIP-1β (*n* = 32), MMP1 (*n* = 32), and IFN-γ (*n* = 32)] were analyzed by paired *t*-test. *p < 0.05; ***p < 0.001; ****p < 0.0001.

Senescent cells accumulate with aging and lead to the release of SASP-related factors including pro-inflammatory cytokines (IL-1α, IL-17, and IL-6), chemokines (IL-8), and proteases (MMP-1, MMP-1α, and MMP-1β). These SASP-related factors play critical roles in aging-related inflammation, diseases, and morbidity (37–39). Therefore, to check whether NK cell infusion decreased systematic levels of SASP-related factors, we measured cytokine levels in plasma collected before and after NK cell infusion. We found lower levels of key SASP-related factors, including IL-6, IL-8, IL-1α, IL-17, MIP-1α, MIP-1β, and MMP1, in the plasma after NK cell infusion, whereas IFN-γ, a non-SASP-related factor, was not continuously significantly altered (**Figure 5**). These results indicated that NK cell infusion could attenuate the accumulation of SASP-related factors and improve CD4^+^ and CD8^+^ T cells’ activities.

### Senescent T cells and exhausted T cells have no significant changes in the control group

**Figure 6:**
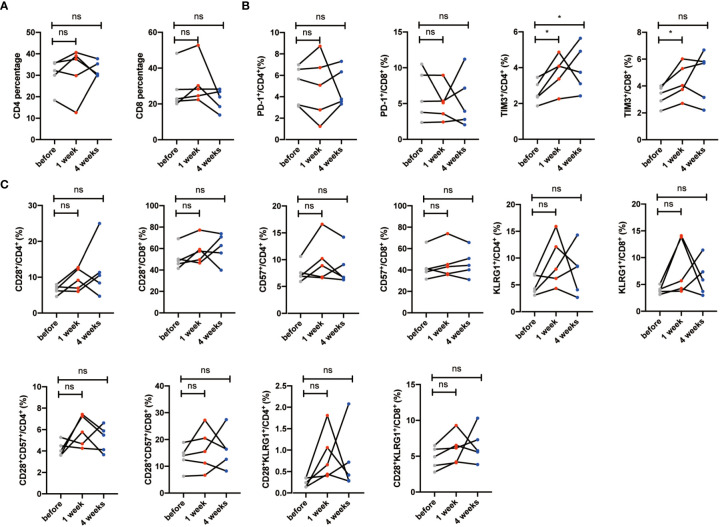
Senescent T cells and exhausted T cells have no significant changes in the saline group. **(A)** Percentages of CD28^-^, CD57^+^, KLRG1^+^, CD28^-^CD57^+^, and CD28^-^KLRG1^+^ T cells were analyzed by flow cytometry on the 1 week and 4 weeks after saline injection. **(B)** Percentages of PD-1^+^ and TIM-3^+^ T cells were analyzed by flow cytometry on the 1 week and 4 weeks after saline injection. Each marker has detected five volunteers. *p < 0.05. ns, no signifcant.

We had known that the percentages of senescent T cells and exhausted T cells decreased significantly after NK cell infusion. To further validate the relationship between NK cell infusion and T-cell reduction, we obtained five volunteers who were injected with normal saline (**Supplementary Table 1**). We revealed that total CD4^+^ and CD8^+^ T-cell percentages were not significantly changed after saline injection (**Figure 6A**). Exhausted T-cell percentages such as PD-1+ and TIM3+ T cells did not decrease after normal saline injection, neither did senescent T-cell populations such as CD28^-^, CD57^+^, KLRG1^+^, CD28^-^CD57^+^, and CD28^-^KLRG1^+^ T cells (**Figures 6B, C**). Lastly, we explored the data in the larger cohort to assess how much power that these five subjects achieve reductions in senescent T cells (**Supplementary Table 3**); in order to ruled out the hypothesis that normal saline can also cause changes in senescent cell.

### PostNK cells kill senescent CD4^+^ T cells but not normal CD4^+^ T cells

**Figure 7:**
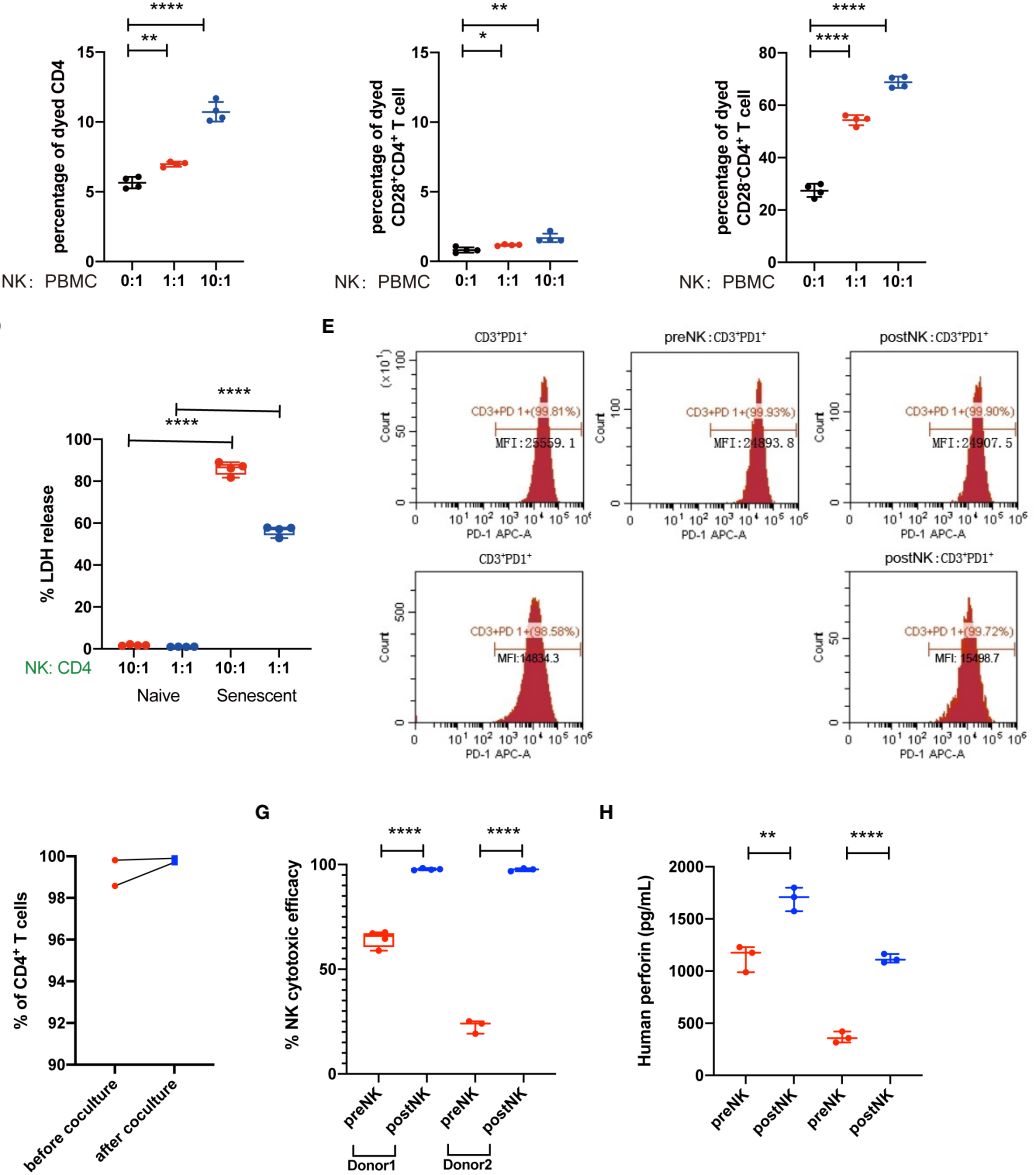
NK cells kill senescent CD4^+^ T cells but not normal CD4^+^ T cells. **(A)** Representative figure showed the percentages of dead CD4^+^ T cells. **(B)** Percentages of dead CD4^+^ T cells as indicated in **(A)**. **(C)** Representative figure showed the percentages of dead CD28^+^CD4^+^ T cells. **(D)** Percentages of dead CD28^+^CD4^+^ T cells as indicated in **(C)**. **(E)** Representative figure showed the percentages of dead CD28^-^CD4^+^ T cells. **(F)** Percentages of dead CD28^-^CD4^+^ T cells as indicated in **(E)**. **(G)** CD28^+^CD4^+^ T cells and CD28^-^CD57^+^CD4^+^ T cells were sorted from PBMC and co-cultured with NK cells, the effects of NK cells in T cells were detected by cytotoxicity assay. **(H)** The LDH release level of the CD28^+^CD4^+^ T-cell group and CD28^-^CD57^+^CD4^+^ T-cell group as indicated in **(G)**. *p < 0.05; **p < 0.01; ****p < 0.0001.

Lastly, we wonder how NK cell infusion influenced the percentages of senescent T cells. Previous studies have demonstrated that NK cells could kill and clear senescent cells to avoid paracrine effect. Thus, we firstly *in vitro* co-cultured PBMCs with autologous postNK cells (**Supplementary Figure 2**), and the percentages of dead CD28^-^ and CD28^+^ CD4^+^ T cells were detected. We found that the percentages of dead CD4^+^ T cells increased after postNK cell adjunction. While causing slight increase of CD28^+^CD4^+^ T cells, postNK cell adjunction dramatically led to increased ratio of dead CD28^-^CD4^+^ T cells (**Figures 7A–C**), critically contributing to increased dead CD4^+^ T cells. Furthermore, instead of total PBMCs, we used CD28^-^CD57^+^CD4^+^ or CD28^+^CD4^+^ T cells to co-culture with autologous postNK cells (**Supplementary Figure 2**) in a Nonradioactive Cytotoxicity assay (Pro-mega). Results showed that postNK cell adjunction specifically killed CD28^-^CD57^+^CD4^+^ T cells other than CD28^+^CD4^+^ T cells in a dose-dependent way (**Figure 7D**). When CD3^+^PD1^+^ T cells were co-cultured with preNK or postNK at an E:T cell ratio of 10:1, no LDH activity were detected after 20 h of incubation (data not shown). Also, NK cell adjunction had little effect on PD1 expression on exhausted T cells (**Figures 7E, F**). postNK cells showed significant improved cytotoxicity against K562 and perforin secretion (**Figures 7G, H**). Nevertheless, IFNγ was detected only in a co-culture sample of preNK cells of donor 1 with K562 at an average concentration of 412.5 pg/ml by ELISA.

### Phenotypic characterization of preNK and postNK cells

**Figure 8:**
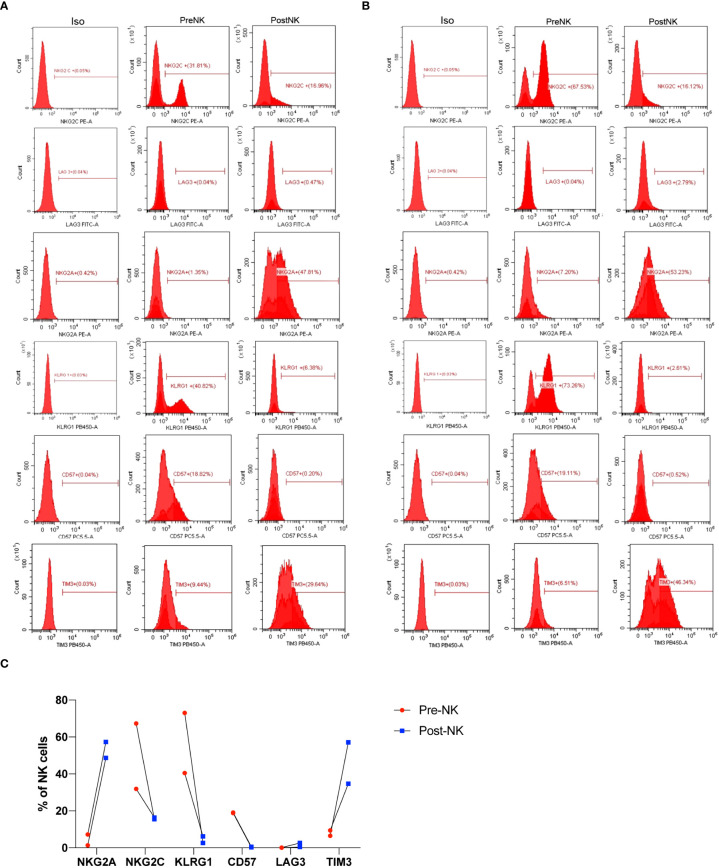
Phenotypic characterization of preNK and postNK cells. **(A)** preNK and postNK cells of donor 1 were used for flow cytometry analysis of NKG2A, NKG2C, LAG3, KLRG1, CD57, and TIM3. **(B)** preNK and postNK cells of donor 2 were used for flow cytometry analysis of NKG2A, NKG2C, LAG3, KLRG1, CD57, and TIM3. **(C)** The alteration trend of indicated receptors.

In order to better understand why the infusion of expanded NK cells showed significant improvement of T senescence and exhaustion, we further compared phenotypic variation of NK cells after *in vitro* expansion procedure (**Figures 8A–C**). preNK cells of two individuals mainly expressed NKG2C, KLRG1, and CD57, while having a much lower level of NKG2A. LAG3 was barely expressed in both preNK and postNK cells. After the expansion procedure, postNK cells had a lower expression of NKG2C, CD57, and KLRG1, but a higher expression of NKG2A and TIM3. Additionally, postNK cells of different individuals showed a more similar expression pattern of indicated receptors than their preNK counterparts.

## Discussion

Here, we firstly uncovered that a single administration of autologous NK cells in middle-aged healthy individuals not only significantly decreased peripheral senescence and exhaustion T cells, but also lowered the secretions of key SASP-related factors, which is another critical factor highly related to aging. It is well documented that the immune system undergoes a progressive decline and deterioration with aging, which, in turn, results in increased incidence and severity of infections, impaired response to vaccines, and accelerated development of cancer. Therefore, developing efficient measures to eliminate or at least alleviate the phenomenon of immune dysfunction may theoretically and practically restore or improve the protective function of the immune system.

Of note, autologous NK cell infusion was proven to be safe for human use, because no adverse events were observed (**Supplementary Table 2**). After autologous NK cell infusion, CD28^-^, CD57^+^, and KLRG1^+^ subsets were significantly reduced within CD4^+^ and CD8^+^ T cells. Meanwhile, similar changes were observed in the CD28^-^CD57^+^ and CD28^-^KLRG1^+^ CD4^+^ and CD8^+^ T subsets. Furthermore, T-cell exhaustion was also alleviated significantly as indicated by reduced levels of CD4^+^PD-1^+^, CD4^+^TIM-3^+^, CD8^+^PD-1^+^, and CD8^+^TIM-3^+^ (**Figures 2**, **3**). However, no significant differences of T-cell senescence and exhaustion index were observed after normal saline treatment (**Figure 6**). In addition, our preliminary data also revealed that autologous NK cell infusion was unlikely able to affect the percentage of Th1, Th2, Th17, and Treg in CD4^+^ T cells (data not shown). Accordingly, more examinations have to be carried out in the future to address this issue. Although one infusion was performed here, the effects were not transient but continued for at least 4 weeks, and much longer-term effects should be evaluated in the future to further characterize the pharmacodynamics of NK cell infusion.

We also studied whether NK cell-mediated alleviation of T-cell senescence and exhaustion was restricted to the sex of subjects. There was no significantly different trend in both male and female groups, strongly implying that the effects induced by NK cell infusion are independent with sex. Additionally, NK cells generated from fresh PBMCs were shown to significantly affect more indices of senescent and exhausted T cells, indicating that fresh PBMCs are better for starting NK cell amplification. Given that cryopreservation is a widely used technique for long-term preservation of cell products such as commercial CAR-T cell and stem cell products, we believed that more subjects should be recruited and studied in the future to evaluate the effect of PBMC cryopreservation on adaptive NK cell-mediated immuno-modulatory improvement.

Cellular senescence is one of the 14 hallmarks of aging. Senescent cells play critical roles in age-related immune dysfunction and inflammation, because they release dangerous signals to stimulate immune cells and usually these stimulations are chronic, which, in turn, results in T-cell senescence and exhaustion. Senescent cells also share an important feature in SASP. Previous findings have reported that NK cells could eliminate senescent cells. Here, we found that NK cell infusion significantly decreased the secretions of key SASP-related components including IL-6, IL-1α, IL-8, MIP-1α, MIP-1β, and MMP1, but not IFN-γ, which was a non-SASP-related factor (**Figure 5**). These findings strongly suggested that NK cell infusion alleviated cell senescence during aging. Senescent cells play critical roles in age-related immune dysfunction and inflammation, because they release danger signals to stimulate immune cells and usually these stimuli are chronic, which, in turn, result in T-cell senescence and exhaustion. In short-term co-culture, autologous expanded NK cells were found to affect senescence of T cells, but not the exhaustion (**Figures 7A–F**), mechanically elucidating that NK cell infusion-related effects on senescent T cells were probably due to their capacity to eliminate senescent cells *in vivo*. In terms of T-cell exhaustion, longer co-cultures should be performed in the future to further testify whether NK cells directly affect T-cell exhaustion or not. It is known that T-cell exhaustion is reversible (40), and immune checkpoint inhibitors and antiretroviral therapy were reported to reverse the dysfunction and phenotype of exhausted T cells under the circumstance of chronic viral infection (41, 42). After these treatments, exhausted CD8+ T cells gained cytotoxic activity and had a lower level of PD-1. Accordingly, our findings of decreased T-cell exhaustion could be an indirect effect due to significant alleviation of the senescence and its associated chronic inflammation by NK cell infusion.

Notably, expanded NK cells were found to have enhanced cytotoxic activity and perforin secretion against K562 (**Figures 7G, H**). Nevertheless, these expanded NK cells expressed a lower level of NKG2C and CD57 but a higher level of NKG2A and TIM3 (**Figure 8**), indicating that they were not fully mature (43, 44). Due to the fact that the culture conditions here are patented, it is hard to fully elucidate the underlying mechanism. Nevertheless, Lieberman et al. also reported that by using a K562 cell line expressing membrane-bound IL-15 and 4-1BB ligand with high-dose soluble IL-2, similar phenotypically immature and functionally pleiotropic NK cells were generated (45). They suggested that these subsets of NK cells were preferred to be expanded and may persist better *in vivo*. Other stimuli, including the presence of IL-12 and IL-18 in culture, were shown to induce NKG2A expression (46, 47). Moreover, KLRG1, one of inhibitory receptors to negatively regulate NK cell function (48), was also found to be significantly inhibited in expanded NK cells (**Figure 8**), implicating its roles to contributing to enhanced function of NK cells post expansion. After infusion, whether and how these immature expanded NK cells will sustain or change in either phenotypic or functional ways should be addressed to better our understanding of their *in vivo* mode of action.

## Conclusions

In conclusion, the present study originally uncovered the effect of NK cell infusion on T-cell dysfunction and cellular senescence in middle-aged healthy individuals. Our findings showed that autologous NK cell administration was an efficient method to significantly alleviate T-cell senescence and exhaustion, as well as key components of SASP. postNK cells showed improved cytotoxic activity and can specifically and directly recognize and eliminate senescent T cells. Also, different expression patterns of activating and inhibitory receptors including NKG2C, NKG2A, KLRG1, LAG3, CD57, and TIM3 were observed after *in vitro* expansion procedure, probably contributing to their functional alteration. Our data importantly indicated that aging, at least for the immune system, could be manipulated towards a younger direction by the transfer of autologous NK cells. Further exploration should focus on the molecular mechanism that autologous NK cells influence T-cell senescence and exhaustion. Also, further investigation into these senescent and exhausted T-cell populations, their origin, and their function in immunologic pathologic conditions will greatly promote clinical use of NK immunotherapy.

## Data availability statement

The raw data supporting the conclusions of this article will be made available by the authors, without undue reservation.

## Ethics statement

This study was reviewed and approved by ClinicalTrials.gov identifier: ChiCTR-OOh-17011878. The patients/participants provided their written informed consent to participate in this study.

## Author contributions

HY, BL and XW were responsible for the conception of the article and project. XT, BD and AZ were responsible for analyzing data and writing the article. XH, YZ, DW, DL, XD, XZ, YL, YX, JJC, WZ and LZ helped us collect blood sample. CG helped us review the article. JQC helped us in the revision process. HY, BL and XW critically revised the entire manuscript and approved the final version. All authors contributed to the article and approved the submitted version.

## Funding

This study was supported by National Natural Science Foundation of China, grant number 81470915; Construction of Shanghai Innovation Center, National Human genetic resources sharing service platform, project number YCZYPT[2017]02; Cell Therapy Special Project of Shanghai Changzheng Hospital, Number CTSP201702; and Scientific Collaborative Project between Shanghai Origincell Medical Technology and Institute Pasteur of Shanghai about Assessment of Immune System Status in Adults.

## Conflict of interest

Authors AZ, XH, DW, XZ and HY are employed by Shanghai Origincell Medical Technology Co., Ltd. Author JQC is employed by Shanghai Affinity Biopharmaceutical Co., Ltd.

The remaining authors declare that the research was conducted in the absence of any commercial or financial relationships that could be construed as a potential conflict of interest.

## Publisher’s note

All claims expressed in this article are solely those of the authors and do not necessarily represent those of their affiliated organizations, or those of the publisher, the editors and the reviewers. Any product that may be evaluated in this article, or claim that may be made by its manufacturer, is not guaranteed or endorsed by the publisher.
