# Supplementary material for: Characterization of age-related immune features after autologous NK cell infusion: Protocol for an open-label and randomized controlled trial
Source: Front Immunol. 2022 Sep 29;13:940577. doi: 10.3389/fimmu.2022.940577 (PMC9562930; doi:10.3389/fimmu.2022.940577)
Supplement: Supplementary file 1 [file Table_1.docx]

| **Supplementary Table 1: Baseline characteristics** | |
| --- | --- |
| **Autologous NK infusion Group** | |
| **Subjects** (n) | 32 |
| **Age range at infusion** (years) | 45-55 |
| **Weight** (kg , mean±SD) | 64.01±10.95 |
| **Sex** |  |
| Male (n) | 15 |
| Female (n) | 17 |
| **Total Cell No.** (×10^9) |  |
| Mean±SD | 5.36±0.69 |
| 95% CI | 5.06-5.65 |
| Min~Max | 4.20-6.70 |
| **NK cell purity** (%) |  |
| Mean±SD | 54.73±15.84 |
| 95% CI | 48.03-61.42 |
| Min~Max | 28.0-86.0 |
| **Expansive fold increase of NK cells (**Mean±SD) | 351.15±266.76 |
| **Saline group** | |
| **Subjects** (n) | 5 |
| **Age range at infusion** (years) | 46-50 |
| **Weight** (kg , mean±SD) | 64.40±6.80 |
| **Sex** |  |
| Male (n) | 3 |
| Female (n) | 2 |
