# Supplementary material for: Characterization of age-related immune features after autologous NK cell infusion: Protocol for an open-label and randomized controlled trial
Source: Front Immunol. 2022 Sep 29;13:940577. doi: 10.3389/fimmu.2022.940577 (PMC9562930; doi:10.3389/fimmu.2022.940577)
Supplement: Supplementary file 2 [file Table_2.docx]

| **Supplementary Table 2: Adverse events after autologous NK cell infusion.** | | |  | |  |
| --- | --- | --- | --- | --- | --- |
| **Adverse event** | Cases | Time | | Gender | |
| **Skin rashes** | 0 | 0 | | 0 | |
| **Local bleeding, infection** | 0 | 0 | | 0 | |
| **Fever and chills** | 0 | 0 | | 0 | |
| **Infection** | 0 | 0 | | 0 | |
| **Difficult breathing** | 0 | 0 | | 0 | |
| **Nausea and vomiting** | 0 | 0 | | 0 | |
| **Low blood cell count** | 0 | 0 | | 0 | |
| **Agrypnia** | 1 | 1^st^ week | | F | |
| **Dizziness** | 1 | 1-2 weeks | | F | |
| **Fatigue** |  |  | |  | |
| mild | 1 | 1^st^ week | | F | |
| media | 1 | 1-2 weeks | | F | |
| severe | 0 | 0 | | 0 | |
| **others** | **0** | **0** | | **0** | |
