# Supplementary material for: Characterization of age-related immune features after autologous NK cell infusion: Protocol for an open-label and randomized controlled trial
Source: Front Immunol. 2022 Sep 29;13:940577. doi: 10.3389/fimmu.2022.940577 (PMC9562930; doi:10.3389/fimmu.2022.940577)
Supplement: Supplementary file 3 [file Table_3.docx]

**Supplementary Table 3: Power calculation of saline group**

| CD4 | 0.5815 |
| --- | --- |
| CD8 | 0.4799 |
| CD4+PD-1+ | 0.411 |
| CD8+PD-1+ | 0.3529 |
| CD4+CD28- | 0.9474 |
| CD8+CD28- | 0.7311 |
| CD4+CD57+ | 0.9152 |
| CD8+CD57+ | 0.5481 |
| CD4+TIM3+ | 0.5605 |
| CD8+TIM3+ | 0.6982 |
| CD4+KLRG1+ | 0.7617 |
| CD8+KLRG1+ | 0.9465 |
| CD4+CD28-CD57+ | 0.9511 |
| CD8+CD28-CD57+ | 0.8843 |
| CD4+CD28-KLRG1+ | 0.949 |
| CD4+CD28-KLRG1+ | 0.7342 |
