# Supplementary figures and images for: Characterization of age-related immune features after autologous NK cell infusion: Protocol for an open-label and randomized controlled trial
Source: Front Immunol. 2022 Sep 29;13:940577. doi: 10.3389/fimmu.2022.940577 (PMC9562930; doi:10.3389/fimmu.2022.940577)

**Supplementary Figure 1. Representative phenotype of NK cells**

（A）


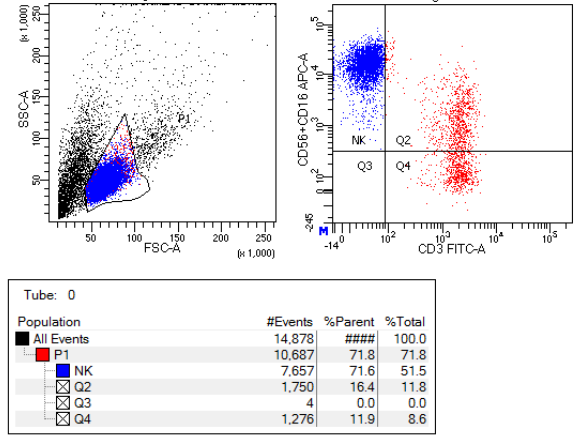


（B）


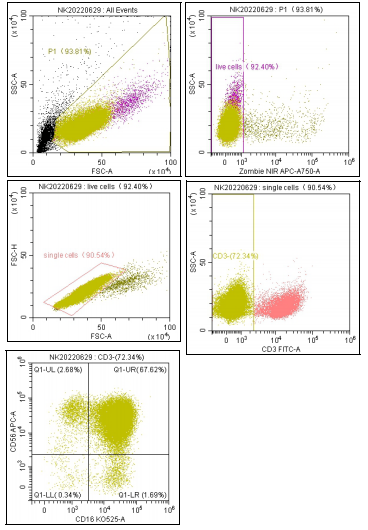

Supplement: Supplementary file 4 [file DataSheet_1.docx]

**Supplementary Figure 2**

（A）


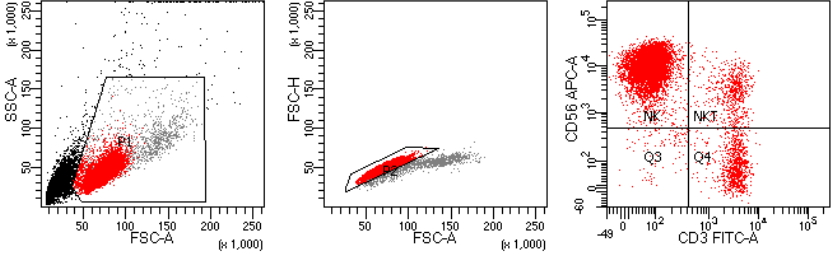


（B）


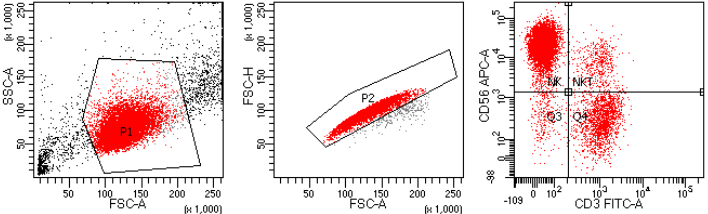

Supplement: Supplementary file 5 [file DataSheet_2.docx]
